# Progress in biostimulation-based remediation of TPH-contaminated soils: a comprehensive review

**DOI:** 10.7717/peerj.19991

**Published:** 2025-09-08

**Authors:** Yuanyuan Li, Jinqiang Yang, Yaru Song, Mingli Wei

**Affiliations:** 1 School of Civil Engineering, Qingdao University of Technology, Qingdao, Shandong, China; 2 State Key Laboratory of Geomechanics and Geotechnical Engineering, Institute of Rock and Soil Mechanics, Chinese Academy of Sciences, Wuhan, Hubei, China; 3 Jiangsu Institute of Zoneco Co., Ltd., Yixing, Jiangsu, China

**Keywords:** TPH contaminated soil, Petroleum contaminated soil, Biostimulation, Microbial remediation, Soil pollution

## Abstract

**Background:**

Petroleum pollution in soils poses a significant global environmental threat, primarily due to increased industrial activities and oil spills. Biostimulation, a bioremediation strategy that enhances indigenous microbial activity through nutrient amendment, has gained prominence for its sustainability and effectiveness.

**Methods:**

This review consolidates findings from 276 peer-reviewed studies published between 2000 and 2025, covering the application of organic amendments, mineral nutrients, and biochar. It further analyzes microbial community responses, degradation efficiency, and synergistic treatment outcomes under varied environmental conditions.

**Results:**

Biostimulants such as compost, nitrate, and phosphate significantly enhance microbial degradation of total petroleum hydrocarbons (TPHs), especially when used in combination. Biochar improves soil aeration and supports functional gene expression. Integrated treatments demonstrate superior removal efficiencies compared to single-agent approaches.

**Conclusion:**

Biostimulation is a promising, scalable solution for TPH-contaminated soils. However, optimization of nutrient ratios, consideration of site-specific variables, and real-time monitoring systems are essential for effective field deployment.

## Introduction

Due to the widespread use of petroleum as a resource required for human industry and the extensive building of petroleum wells and other infrastructure for petroleum extraction to fulfill demand, petroleum pollution has grown to be an international environmental issue. Tank breaches and transportation mishaps consequently frequently transpire, severely impairing ecological habitats, including soil fertility and other attributes (Mohanta, Pradhan & Behera, 2023).

Among the existing soil remediation technologies, physical and chemical remediation, although able to alleviate pollution to a certain extent, are generally characterized by high cost, complex operation, and the possibility of secondary pollution. In contrast, biostimulation, as an emerging bioremediation technology, has gradually received widespread attention owing to its unique advantages. First, the biostimulation method is low cost and easy to operate, making it very suitable for large-scale promotion. Second, as a microbial remediation method, the technique is environmentally friendly, free of secondary pollution, and highly sustainable (Chunyan et al., 2023). Finally, owing to the wide range of materials available for biostimulation, they can be customized to remediate different types of petroleum contaminants and improve the remediation effect (Robichaud et al., 2019). In addition, the two primary bioremediation techniques are bioaugmentation (BA) and biostimulation (BS). The main approach to bioaugmentation entails introducing external microorganisms into a contaminated site (Ajona & Vasanthi, 2021; Safdari et al., 2018). In contrast, the primary purpose of biostimulation is to improve the metabolic activity of naturally occurring microbial communities by adding specific biostimulants, which helps to remediate soils contaminated with petroleum hydrocarbons (Xu et al., 2020; Fenibo et al., 2019; Tahmasbizadeh et al., 2025). In many cases, the bioaugmentation method needs to adapt to the inoculation environment owing to the addition of exogenous strains. Achieving the same remediation effect often requires longer remediation time; in some cases, the remediation effect is not as good as that of the biostimulation method, Table 1 shows the remediation effect of BS and BA on total petroleum hydrocarbons (TPH) under different soil conditions (Safdari et al., 2018; Wu et al., 2019; Yaman, 2020; Ying et al., 2022).

**Table 1:** Comparison of TPH degradation rates between the biostimulation and bioaugmentation methods.

Serial number	Type of pollutant	TPH concentration (mg/kg)	Added substance	Processing cycle	Maximum TPH removal rate	Reference
1	Petroleum	44,600	Acinetobacter	70 d	34%	Wu et al. (2016)
			(NH_4_S)_2_O_4_ KH_2_PO_4_		60%	
2	Diesel oil	20,000	*Pseudomonas aeruginosa Bacillus subtilis*	60 d	65.8%	Safdari et al. (2018)
			KH_2_PO_4_ NaHPO_4_ NH_4_Cl NaCl		84.7%	
3	Petroleum	15,233	Petroleum degrading flora	60 d	17.87%	Wang et al. (2021b)
			KNO_3_		44.77%	
4	Petroleum	19,800	Petroleum degrading flora	84 d	13.9%	Wu et al. (2019)
			NH_4_NO_3_ KH_2_PO_4_		28.3%	
5	Petroleum	1,674	Petroleum degrading flora	63 d	41%	Yaman (2020)
			NH_4_Cl KH_2_PO_4_		66%	

Although several reviews have addressed bioremediation techniques, including bioaugmentation and natural attenuation, this review provides a distinct and updated perspective by specifically focusing on recent advances in biostimulation-based remediation of petroleum-contaminated soils. Unlike earlier studies that primarily discussed traditional nutrient addition, this work systematically incorporates the latest developments in biostimulant materials, with a strong emphasis on emerging technologies such as nanomaterial-enhanced soil amendments (Sayed et al., 2022), genetically engineered microorganisms (GEMs) that exhibit enhanced hydrocarbon degradation pathways (Sayed et al., 2021), and the integration of artificial intelligence (AI)-driven biosensing technologies for real-time monitoring and optimization of field conditions (Sayed, Baloo & Sharma, 2021). Furthermore, this review distinguishes itself by bridging the gap between laboratory-scale findings and practical field-scale implementation. It highlights not only the functional mechanisms of organic, inorganic, and hybrid biostimulants, but also their impact on microbial community structure, functional gene expression, and degradation kinetics. The novelty of this review lies in its interdisciplinary approach—consolidating insights from microbial ecology, soil chemistry, and environmental monitoring technologies. thereby offering a comprehensive and scalable framework for petroleum remediation strategies in complex and heterogeneous environments. Moreover, this work addresses critical knowledge gaps by contextualizing synergistic applications (*e.g*., biochar + nutrient combinations) and real-time sensing tools within the broader context of sustainable soil remediation, supported by recent peer-reviewed findings (Oldani et al., 2023). Overall, this review not only captures the current state of biostimulation but also proposes future directions that align with global sustainability goals and field-level remediation efficacy.

Therefore, compared to the bioaugmentation method, its practical engineering applications are stronger. Despite the significant advantages of the biostimulation method in theory, it still faces challenges in practical applications, such as the selection of materials, improvement of remediation efficiency, and shortening of the remediation cycle. Therefore, it is of great significance to increase research on biostimulation methods and optimize the remediation process to solve the problem of oil-contaminated soil.

## Survey methodology

Given the growing body of literature on biostimulation as a remediation strategy for TPH-contaminated soils, we conducted a comprehensive literature review to map key biostimulant types, their mechanisms, environmental impacts, and field-scale effectiveness. The primary objective of this review was to consolidate scattered findings, identify knowledge gaps, and propose future directions for the optimized application of biostimulation in real-world scenarios. Unlike meta-analyses or systematic reviews that focus on quantifying outcomes, our approach was exploratory, enabling a broad conceptual overview of emerging trends, material diversity, microbial interactions, and technological integration.

To assemble a comprehensive and unbiased literature base, we used four major academic databases: Web of Science, Scopus, ScienceDirect, and Google Scholar. Search terms included: *“TPH-contaminated soil”*, *“biostimulation”*, *“bioremediation”*, *“organic amendments”*, *“petroleum hydrocarbons”*, and *“nutrient-based soil remediation.”* No time restriction was imposed to ensure coverage of both foundational and recent studies, although most literature reviewed spanned from 2000 to 2024.

The search process involved three screening stages:

- Initial count: The combined search across all databases yielded 1,267 articles matching the base search criteria.
- Duplicate and scope-based exclusion: Using Mendeley for reference management, 278 duplicate entries were removed. We then screened titles and abstracts to eliminate non-English articles, conference abstracts, unrelated microbial studies, and remediation approaches not focused on biostimulation or TPH.
- Full-text screening: Of the remaining 418 articles, 142 were excluded for lacking methodological clarity, quantitative data on TPH degradation, or clear identification of biostimulants. The final set included 276 peer-reviewed articles and review articles, forming the primary dataset.

Articles were included if they met the following criteria:

(1) Peer-reviewed, English-language publications;
(2) Full-text available;
(3) Addressing biostimulation for TPH remediation in soil systems;
(4) Presenting experimental, field, or comparative review data.

We also performed citation chaining (both forward and backward) to include influential articles referenced in high-citation reviews and experimental studies. In total, this allowed for both horizontal (across methods) and vertical (within biostimulant categories) coverage of the field.

This structured and iterative methodology enabled us to provide a comprehensive and current synthesis of biostimulation research, explore emerging trends such as biochar-mineral synergies, and identify critical gaps related to field implementation, microbial monitoring, and engineering scalability.

## Main stimulation mechanisms of biostimulants

### Improvement of the nutritional environment

The core mechanism of the biostimulation method is to provide nutrients to the microorganisms, and the lack of nutrients will have a great impact on the microbial degradation efficiency. Carbon, nitrogen, and phosphorus are crucial for the growth and development of native microbial bacteria that can break down substances, as they use petroleum hydrocarbons as a carbon source for their growth (Lan et al., 2019; Shahi et al., 2016; Anekwe & Isa, 2024). Indigenous microorganisms in the soil must constantly utilize nitrogen in the soil in order to maintain their metabolism. Moreover, soil microorganisms need a specific amount of potassium to activate the necessary endoenzyme in the initial growth phases, allowing them to adjust to the contaminated environment and use the oil as a carbon source. The lack of phosphorus in the soil inhibits the biomass of soil microorganisms and their efficiency in degrading organic pollutants, which is detrimental to the biodegradation of oil. Thus, the deficiency of nutrients like nitrogen (N), phosphorous (P), and potassium (K) significantly restricts microbial growth, making nutrient addition crucial for improving the breakdown of petroleum in polluted soils (Wu et al., 2016; Deng et al., 2025). Biostimulation can enhance the proliferation and growth of microorganisms by introducing biostimulants into the soil, which supply various nutrients to these microorganisms. For example, some manures such as plant wastes not only have chemical forms of nitrogen, mainly ammonium nitrogen, amino acids, or amides that are easily hydrolyzed, but are also rich in nutrients such as N and P, which can effectively improve the content of nutrients in the soil (Liu et al., 2020; Tsai et al., 2025). Moreover, other biostimulants like biochar can supply nutrients through their functional groups, while mineral fertilizers and plants can offer nutrients to microorganisms, thereby promoting the growth of native microbial degrading bacteria to enhance the TPH degradation rate.

The efficiency of microbial hydrocarbon degradation is strongly influenced by nutrient availability, particularly the carbon: nitrogen: phosphorus (C:N:P) ratio. Studies report optimal biodegradation when the ratio is maintained near 100:10:1, although ranges like 100:5:1 and 100:15:1 have also shown effectiveness depending on soil conditions (Shahi et al., 2016; Cheng, Zhou & Yu, 2019; Wu et al., 2019). Ammonium nitrate (NH_4_NO_3_) and monopotassium phosphate (KH_2_PO_4_) were added to petroleum hydrocarbon-contaminated soil to achieve a carbon: nitrogen: phosphorus (C:N:P) ratio of 100:10:1. This nutrient amendment resulted in a total petroleum hydrocarbon (TPH) degradation rate of 28.3% after 12 weeks, indicating effective stimulation of indigenous petroleum-degrading microbial communities. After 12 weeks the TPH degradation reached 28.3% showing that nitrogen and phosphorus addition had enriched the microbial composition toward petroleum-hydrocarbon-degrading conditions. The scientific community describes 100:10:1 as an ideal ratio between carbon nitrogen and phosphorus to conduct biostimulation strategies (Cheng, Zhou & Yu, 2019). The proper C/N/P ratio required for soil remediation differs between contaminated soils because diverse properties lead to different microbial community compositions. Research conducted by Wang et al. (2021b) determined KNO_3_ as the best biostimulant when added with petroleum hydrocarbon contaminated soils. These researchers found the optimal C/N ratio of 100:20 for petroleum hydrocarbon remediation while it is possible that the required nitrogen levels for microbial activity vary based on soil region. Microbial degradation depends heavily on controlling nutrient ratios in biostimulation methods because different soil environments might need dissimilar C/N/P ratios.

### Improve the physical and chemical properties of the soil

The soil’s porosity greatly affects how microbes break down organic matter, as smaller pores can hinder the interaction between microbes and organic material (Akbari & Ghoshal, 2015). Moreover, when petroleum hydrocarbons are present in the soil, they tend to obstruct it, thereby diminishing the soil’s ability to retain water and allow air circulation, both of which are essential for the growth and development of microorganisms (Abosede, 2013). The addition of biostimulants can be effective in improving the porosity of the soil. For instance, Huang et al. (2019) incorporated rice straw into soil contaminated with petroleum, as the straw’s hollow structure could enhance soil aeration. This characteristic might also render the amended soil more conducive to microbial habitats for the degradation of TPHs. Similarly, Akpokodje & Uguru (2019) remediated petroleum hydrocarbon contaminated soil using organic compost and organic soap, it was found that both significantly improved soil porosity and specific gravity, which enhanced the remediation of petroleum hydrocarbons. In another approach, Lv et al. (2023a) utilized biochar aerogel material prepared at high temperatures as remediation material. The biochar aerogel’s hierarchical pore structure and extensive specific surface area enhanced soil porosity and created an ideal environment for microorganisms to thrive and multiply. Additionally, incorporating biochar aerogel boosted the population of petroleum hydrocarbon-degrading bacteria primarily by stimulating microbial activity. Saeed et al. (2021) added biochar to petroleum hydrocarbon contamination, which increased the soil moisture content by 20% comparing to the contaminated soil without biochar.

Soil parts hydrogen (PH) is also important for the survival and development of microorganisms, and an appropriate PH is favorable for the growth and development of microorganisms. Many material-based biostimulants derive their PH-regulating ability from their own PH under natural conditions, so it is important to choose the appropriate biostimulant for soil conditions with different PH (Lee et al., 2022b). However, for plant-based biostimulation, its ability to self-regulate inter-root pH, water, oxygen, and effective nutrients provides suitable conditions for microorganisms to survive and has a favorable effect on microbial degradation of petroleum hydrocarbons (Kotoky, Rajkumari & Pandey, 2018).

### Increase microbial diversity

The degradation efficiency of a single petroleum hydrocarbon-degrading bacterium is often inferior to that of a system constructed using multiple petroleum hydrocarbon-degrading bacteria (Jia, Li & Deng, 2023). In indigenous petroleum hydrocarbon-contaminated sites, indigenous petroleum hydrocarbon-degrading bacteria do not appear as a single species but as a combination of microbial communities for the synergistic degradation of petroleum hydrocarbons. As a result, the variety of microbial communities greatly influences the effectiveness of degradation. Incorporating biostimulants can boost the variety of bacteria that break down petroleum hydrocarbons and improve the efficiency of their degradation. Shi et al. (2022) added soybean straw, lemon leaves and corn cobs as exogenous stimulants to petroleum hydrocarbon contaminated soil and found that these biostimulants activated the indigenous microbial flora and improved the microbial diversity, and compared the microbial community compositions of different biostimulants were found to be different. Other studies have also demonstrated that different biostimulants activate different indigenous degrading bacteria and produce different microbial community structures (Huang et al., 2019; Zhang et al., 2021b).

The introduction of biostimulants allows activation of native microbial degradation flora together with the ability to transport indigenous petroleum hydrocarbon degrading bacteria to break down petroleum hydrocarbons in contaminated soils. The contaminants found in animal waste introduce numerous microorganisms into crude oil-contaminated soils while delivering essential nutrients through phosphorus and potassium and nitrogen to facilitate microbial development. The microorganisms use hydrocarbons as their carbon source when they break down these compounds while remaining in the presence of oxygen (Douglas et al., 2024). The use of aged refuse as soil amendment brings beneficial petroleum-degrading bacteria that improve petroleum hydrocarbon transformation processes (Chen et al., 2019b).

In addition to enhancing microbial abundance and community diversity, biostimulation significantly contributes to the activation of key functional genes responsible for the degradation of total petroleum hydrocarbons (TPHs). Nutrient amendments, particularly those involving nitrogen- and phosphorus-rich sources, stimulate indigenous microbial communities to express higher levels of catabolic genes. Notably, genes such as *alkB* (alkane monooxygenase), *CYP153* (cytochrome P450 monooxygenase), and *nahAc* (naphthalene dioxygenase alpha subunit) are well-documented markers of hydrocarbon degradation capacity. The *alkB* gene is commonly associated with the aerobic oxidation of mid- to long-chain alkanes, while *CYP153* is involved in terminal hydroxylation reactions, and *nahAc* is essential for the breakdown of polycyclic aromatic hydrocarbons (PAHs) like naphthalene (Zhang, Wu & Ren, 2020; Yao et al., 2024).

Recent metagenomic and qPCR-based studies have confirmed that biostimulants not only alter the structure of soil microbial communities but also induce quantitative increases in gene transcripts related to xenobiotic metabolism. For instance, nutrient enrichment using ammonium nitrate and phosphate fertilizers resulted in a 3–5-fold increase in *alkB* gene abundance, correlating with accelerated TPH removal rates in sandy loam and clay-rich soils. Additionally, the stimulation of nitrogen-fixing bacteria and biosurfactant-producing taxa has been linked to higher expression of genes related to membrane transport and stress tolerance, further enhancing the microbial capacity to adapt to hydrocarbon stress.

Thus, functional gene activation represents a pivotal mechanism through which biostimulation enhances TPH biodegradation, moving beyond simple microbial proliferation to a more functionally enriched, genetically equipped microbial consortium capable of sustained remediation under varied environmental conditions.

#### Functional genes, biofilms, and microbial interactions in TPH degradation

The efficiency of biostimulation in petroleum hydrocarbon remediation is fundamentally driven by the metabolic pathways, gene expression, and cooperative interactions within soil microbial communities. Understanding these molecular and ecological processes provides a mechanistic foundation for optimizing biostimulant design and application.

One of the most critical aspects of microbial hydrocarbon degradation lies in the expression of functional genes encoding catabolic enzymes. Genes such as *alkB* (alkane monooxygenase), *CYP153* (cytochrome P450 monooxygenase), *nahA* (naphthalene dioxygenase), and *rdhA* (reductive dehalogenase) are frequently associated with the degradation of aliphatic and aromatic hydrocarbons. Their transcription is often upregulated in the presence of petroleum hydrocarbons and is strongly influenced by nutrient availability, oxygen content, and biostimulant composition (Wang et al., 2021b; Ren et al., 2022). Quantitative PCR (qPCR) and metagenomic sequencing have revealed that nutrient-rich amendments (*e.g*., poultry manure, compost, or mineral fertilizers) not only increase microbial biomass but also significantly elevate the copy numbers of these functional genes.

In addition to gene-level regulation, biofilm formation plays a pivotal role in maintaining active microbial populations during bioremediation. Biofilms—dense microbial aggregates embedded in extracellular polymeric substances (EPS), enhance survival under environmental stress, improve substrate access, and promote intercellular signaling. Petroleum hydrocarbon degraders such as *Pseudomonas putida* and *Mycobacterium* spp. are known to form robust biofilms on soil particles and biostimulant surfaces like biochar and straw. These structured communities exhibit enhanced resistance to toxic compounds and maintain high local concentrations of degradative enzymes, thereby accelerating hydrocarbon breakdown (Saeed et al., 2021; Shi et al., 2022).

Furthermore, microbial cooperation and consortia dynamics are essential for complete mineralization of complex hydrocarbon mixtures. Diverse microbial taxa exhibit complementary catabolic activities; for instance, one group may oxidize long-chain alkanes, while another converts aromatic intermediates into simpler organic acids. The addition of biostimulants selectively favors the growth of key degraders such as *Rhodococcus*, *Acinetobacter*, *Bacillus*, and *Sphingomonas* and modulates community composition to optimize synergistic degradation. Studies have shown that mixed microbial communities stimulated by plant litter or aged refuse exhibit higher metabolic diversity and resilience compared to single-strain inocula (Zhang et al., 2021b; Chen et al., 2019b).

Elucidating these microbial mechanisms through the study of gene expression, biofilm behavior, and microbial community interactions enables a more predictive and targeted approach to biostimulation. It also supports the design of consortia-based strategies and the use of biostimulants that foster stable, highly active microbial networks for robust petroleum hydrocarbon degradation.

### Quantitative synthesis of TPH removal efficiencies

To enhance the comparative assessment of biostimulation strategies for TPH-contaminated soils, a quantitative synthesis of selected case studies was conducted. Table 2 summarizes the total petroleum hydrocarbon (TPH) removal efficiencies reported across a range of biostimulant types and remediation durations, offering a statistical snapshot of treatment outcomes.

**Table 2:** Summary of TPH removal efficiencies using various biostimulant approaches.

Study	Biostimulant type	TPH removal (%)	Duration (Days)	Notes
Wu et al. (2019)	Mineral Fertilizers (NH_4_NO_3_, KH_2_PO_4_)	28.3%	84	Moderate improvement through N/P addition
Yaman (2020)	BS + BA (NH_4_Cl + KH_2_PO_4_ + microbes)	74.0%	42	Combined treatment outperformed BA or BS alone
Douglas et al. (2024)	Poultry manure	36.0%	60	Higher effectiveness than cow manure
Liu et al. (2018)	Aged refuse (non-sterilized)	89.8%	98	Strong synergy with indigenous microbiota
Seo & Cho (2020)	Compost + Phytoremediation	63.7%	210	Long-term efficiency improvement and increased microbial activity
Zhen et al. (2021)	Rhamnolipid-modified biochar	32.9%	90	Surfactant-modified biochar enabled microbial colonization
Lv et al. (2022)	Biochar + Compost	74.8%	26	Enhanced heavy oil degradation and microbial succession
Bhuyan, Kotoky & Pandey (2023)	NPK + Rhizobacteria	91.0%	60	Highest performance with combined microbial and fertilizer stimulation
Uyizeye, Thiet & Knorr (2019)	Biochar + Compost	62.0%	90	Superior to single-agent treatments

Across nine representative studies, TPH removal efficiencies ranged from 28.3% to 91.0%, with treatment durations spanning 26 to 210 days. The mean removal efficiency was calculated at 61.9%, with a standard deviation of 21.3%, indicating notable variability based on the biostimulant type and site conditions. Single-component biostimulants, such as mineral fertilizers (Wu et al., 2019) and poultry manure (Douglas et al., 2024), showed moderate efficiency (typically below 40%). In contrast, combined strategies, such as NPK with rhizobacteria (Bhuyan, Kotoky & Pandey, 2023) or aged refuse with native microbial communities (Liu et al., 2018), achieved much higher TPH degradation rates, often exceeding 85%.

This synthesis supports the conclusion that multimodal biostimulant treatments consistently outperform single-agent approaches, owing to synergistic effects such as enhanced microbial activation, nutrient cycling, and soil conditioning. It also reinforces the importance of considering remediation duration and site-specific conditions—including nutrient availability and microbial composition, when selecting biostimulation protocols. These findings establish a comparative framework that practitioners and researchers can reference when designing or evaluating bioremediation projects.

### Multi-disciplinary perspectives in biostimulation

While biostimulation has traditionally been approached from a microbial and environmental science perspective, a comprehensive understanding requires integration of ecological, genetic, and engineering dimensions. These interdisciplinary insights can help optimize remediation outcomes, uncover molecular mechanisms, and improve scalability in field applications.

From an ecological standpoint, microbial community dynamics play a critical role in the effectiveness of biostimulation. Biostimulant amendments influence not only microbial abundance but also the structure and succession of microbial consortia involved in hydrocarbon degradation. Studies have shown that petroleum-degrading bacteria, including *Pseudomonas*, *Rhodococcus*, and *Mycobacterium*, exhibit synergistic degradation behaviors when nurtured through biostimulants like compost or biochar (Jia, Li & Deng, 2023; Shi et al., 2022). Moreover, biofilm formation on soil particles or biochar surfaces enhances microbial stability and resistance to environmental stress, promoting more sustained degradation activity in fluctuating field conditions.

At the genetic and molecular level, the efficacy of biostimulation is tightly linked to the activation of specific functional genes. Genes such as *alkB* (alkane monooxygenase), *CYP153* (cytochrome P450 monooxygenase), and *rdhA* (reductive dehalogenase) are critical for the initial oxidation and breakdown of complex hydrocarbons (Ren et al., 2022; Yu et al., 2019). The presence and expression of these genes can be influenced by the availability of nutrients and environmental cues provided by biostimulants. Recent advances in metagenomics, transcriptomics, and qPCR allow researchers to monitor the activity of these degradation pathways and adapt biostimulation inputs to maximize microbial enzymatic responses. This molecular-level insight opens new possibilities for precision bioremediation by tailoring treatments based on gene expression profiles.

From an engineering perspective, biostimulation must account for the geotechnical properties of contaminated soils. Hydrocarbon pollution alters key parameters such as porosity, permeability, cohesion, and shear strength, which influence the applicability of remediation strategies (Zhang et al., 2021a; Yazdi & Sharifi Teshnizi, 2021; Mir Mohammad Hosseini et al., 2019). Engineering inputs are especially valuable when scaling up treatments for field applications, where heterogeneous soil types and hydrodynamic conditions may affect amendment delivery and contaminant mobility. For instance, slow-release nutrient formulations and drip irrigation systems have been developed to regulate nutrient availability while minimizing leaching or over-saturation (Wu et al., 2020). Furthermore, microbial catalysis in biostimulated soils has been shown to contribute urease and carbonate production, which could be harnessed in future soil reinforcement technologies (*e.g*., microbially induced calcite precipitation, MICP) (Salimnezhad, Soltani-Jigheh & Soorki, 2021).

Incorporating ecological, genetic, and engineering perspectives enhances the scientific rigor and practical viability of biostimulation. This multi-dimensional approach not only improves our mechanistic understanding of petroleum hydrocarbon degradation but also facilitates the design of robust, site-specific, and scalable remediation solutions.

## Common types of biostimulants

### Nutrient additives

In the biostimulation process, nutrient additives are the most prevalent form of biostimulants. They primarily enhance the activity of microorganisms by supplying nutrients, thereby increasing the rate at which TPH is degraded. The selection of appropriate biostimulants should be based on the perspective of restoration efficiency, cost, and environmental protection. Appropriate biostimulants can not only meet the requirements of the circular economy, but also reduce the consumption of transportation fuels as well as carbon emissions, which makes it more convenient and quicker to be applied in an actual project.

#### Agricultural residue

(1) The use of agricultural residue as a biostimulant serves not only microbial remediation efforts but also addresses the challenge of agricultural residue disposal. Zhang, Wu & Ren (2020) utilized swine factory wastewater as a nitrogen source for bioremediation and as a substitute for process water, and combined remediation with bran as well as exogenous strains of bacteria, which significantly boosted microbial colonization numbers, and the petroleum hydrocarbon degradation rate under the optimal medium conditions reached 68.27 ± 0.71%. There is a limited amount of research on using natural organic matter derived from plant waste to improve soils contaminated with petroleum. Utilizing it as a biostimulant can greatly enhance soil quality, supply microorganisms with the necessary N, P, and K nutrients vital for their growth and metabolic processes, encourage microbial proliferation, and play a crucial role in the bioremediation of soils contaminated with petroleum (Liu et al., 2020; Yao et al., 2024).

Huang et al. (2019) investigated the remediation of petroleum hydrocarbon-contaminated soils by paddy straw and sawdust, of which the degradation of TPH by paddy straw and mineral fertilizer was 45.2%, while there was no significant difference between sawdust and the control. There was a significant difference in the microbial community structure in different treatments, and the abundance of petroleum degrading bacteria in the soil increased with the addition of rice straw and sawdust. Zhang et al. (2021b) but the TPH degradation rate of the mixed treatment was higher than that of single-litter treatments, possibly due to synergistic effects in phosphorus supplementation, increased enzyme activity (invertase and dehydrogenase), and greater abundance of petroleum hydrocarbon-degrading bacteria in the mixed treatment. showed significant synergistic effects in supplementing effective phosphorus, improving invertase and dehydrogenase and the abundance of petroleum hydrocarbon degrading bacteria was different between the mixed plant litters and single apomictic treatment. Plant litter biostimulants provide microbial nutrients which support degrading bacteria growth while improving soil enzyme activity for petroleum hydrocarbon decomposition. The price for this substance remains inexpensive while its acquisition remains straightforward. The multiple advantages achievable through the mixture of plant wastes prove that numerous combining possibilities exist with promising results expected from these applications.

(2) Utilizing animal manure as a biostimulant represents a sustainable approach to remediating petroleum hydrocarbons. This method not only enhances the efficiency of bioremediation but also aligns with economic and sustainability goals (Adewoyin & Arimoro, 2023; Cunningham et al., 2020; Ruley & Tomor, 2024). Douglas et al. (2024) explored the use of cow and poultry manure to remediate soils contaminated with petroleum hydrocarbons. They discovered that poultry manure was generally more effective, achieving a TPH degradation rate of 36%, compared to 23% for cow manure. Additionally, they observed that increasing the amount of manure led to a rise in the number of heterotrophic microorganisms, which in turn suggested a greater degradation of petroleum. Robichaud et al. (2019) compared different biostimulants and found that horse manure had the highest petroleum hydrocarbons removal efficiency and was more effective in improving soil structure as well as fertility. Utilizing animal manure as a biostimulant proved to be more effective in enhancing the soil’s physicochemical characteristics. Compared with other organic fertilizers, manure can not only provide nutrients for microorganisms but also may contain petroleum hydrocarbon-degrading bacteria, which is more conducive to the remediation of petroleum hydrocarbons.

#### Domestic waste-based nutrient additives

(1) Food waste is a readily available and low-cost type of biostimulant. Xu et al. (2019) used food waste (mainly rice, noodles, vegetables, eggs and meat) from a school cafeteria to make a waste supernatant (FS) to be added to petroleum hydrocarbon contaminated soils. Incorporating FS offered a substantial supply of carbon and nitrogen to the IHD (indigenous hydrocarbons degradation bacteria), facilitating the proliferation of indigenous hydrocarbons degradation bacteria. As a result, over a period of 30 days, the TPH degradation rate reached 49.3%. It was observed that an increased C/N ratio accelerated the growth of IHD, suggesting that a higher C/N ratio may promote dominance of IHD. Liu et al. (2019) used meat bone meal (MBM), a by-product of meat processing, as a novel biostimulant, and found that its petroleum hydrocarbon degradation ability was comparable to that of traditional fertilizers (urea-treated), and MBM-treated soil avoided the usual negative effects of urea on soil pH, and its diesel degradation rate was 91.2% within 99 days under aeration treatment.

(2) Aged refuse serves as an effective soil enhancer, biostimulant, and microbial agent, capable of boosting soil fertility and supporting microbial life, while also containing numerous bacteria that degrade petroleum. Due to the high specific surface area and porosity, excellent physicochemical and hydraulic properties, aged refuse is also a good microbial carrier, which is conducive to the growth and aggregation of indigenous petroleum hydrocarbon-degrading bacteria. Liu et al. (2018) used sterilized and non-sterilized aged refuse to remediate petroleum hydrocarbon contaminated soils, it was found that the TPH removal rate of sterilized mineralized refuse was 74.64% after 98 days, compared to 89.83% of non-sterilized mineralized refuse, due to the synergistic effect of conditioner addition, biostimulation, and bioaugmentation leading to better bioremediation. Chen et al. (2019b) found that after 30 weeks of addition of mineralized refuse of TPH degradation was 63.7%, while the combination of aged refuse and nutrients reached 90.2%.

#### Composting

Compost made from organic waste serves a dual role by supplying essential nutrients and activating indigenous petroleum hydrocarbon-degrading bacteria, thereby enhancing microbial development (Barakwan, 2025), thus enhancing soil TPH removal (Sokač et al., 2022; Rahmeh et al., 2021; Wu et al., 2018). Zhang et al. (2011) used compost made from fresh waste (a mixture of leaves, twigs, and greens) as a biostimulant to be added to petroleum hydrocarbon contamination and found that after 60 d, the PAH concentration decreased by 50.5 ± 14.8%, and the significant rise in gram-positive bacteria in the soil demonstrates that compost supports the growth and development of microorganisms. Liu et al. (2021) explored the impact of inorganic salts, organic composts, and sawdust on soils contaminated with petroleum hydrocarbons. Their findings indicated that composting was the most successful method for enhancing the breakdown of TPH, achieving a degradation rate of 35.7% after 60 days. Compost can not only stimulate microbial communities, but also improve soil physicochemical properties. The advantages of using organic compost are similar to those of animal manure and aged refuse. This dual functionality, nutrient enrichment and microbial inoculation, makes compost an effective biostimulant for accelerating the biodegradation of petroleum hydrocarbons, thereby introducing them to petroleum hydrocarbon-contaminated soil, which enhances the degradation process of these hydrocarbons. It can also improve the physical and chemical properties of soil, which is conducive to the survival of microorganisms. However, this process is more complicated in the production steps.

#### Mineral fertilizers

Mineral fertilizers are used to provide elements such as N, P, and K to microorganisms by adding inorganic salts to petroleum hydrocarbon-contaminated soils, thus stimulating the growth of indigenous microbial-degrading bacteria and increasing the degradation rate of TPH. Common mineral fertilizers include NH_4_NO_3_ and KH_2_PO_4_, and the use of mineral fertilizers such as ammonium and phosphate is a good choice (Cai et al., 2020; Ugbune et al., 2025). Mineral fertilizers are generally most effective when used in combination with other remediation methods, such as microbial inoculation or organic amendments, especially when high TPH concentrations lead to nutrient limitations in later stages of remediation (Safdari et al., 2018; Ou et al., 2025). Yaman (2020) used NH_4_Cl and KH_2_PO_4_ as biostimulants and found that their combination with inoculated microorganisms resulted in higher TPH removal than BS and BA alone, with a degradation rate of 74%.Varjani, Pandey & Upasani (2021) used NH_4_NO_3_ and Na_2_HPO_4_ as biostimulants, it was found that the combination of BS and BA gave the best treatment (93.14 ± 1.75% in 42 d). The use of mineral fertilizers and nitrogen and phosphorus as biostimulants is an effective restoration method. However, when the concentration of TPH to be remediated is high, there is a problem of nutrient deficiency at the later stages of remediation. Therefore, regular supplementation of nutrients in contaminated soil will be more effective for effective degradation of difficult to degrade petroleum hydrocarbons. Wu et al. (2020) compared the use of single application of nutrients and regular supplementation of nitrogen and phosphorus nutrients using a drip irrigation unit as nutrients and the results showed that the drip irrigation unit was more effective after 90 d with a TPH degradation rate of 53.5%. This combination strategy helps overcome the limitations of mineral fertilizers alone by ensuring sustained microbial activity and improved bioavailability of nutrients throughout the remediation process.

The use of mineral fertilizers as biostimulants can provide microorganisms with the required nutrients in a targeted manner, making it easier to control the proportion and amount of nutrients used, provide microorganisms with a suitable nutrient environment, and help microorganisms to use petroleum hydrocarbons as their carbon source, thus improving the efficiency of remediation. Mineral fertilizers can help solve the problem of a lack of nutrients for microorganisms, so they are often used in combination with other remediation methods. For example, in a combined chemical and microbial remediation method, the subsequent growth of microorganisms often results in insufficient N. At this time, through the addition of mineral fertilizers, N, P, and other elements can be targeted to control the microbial nutrient environment, stimulate subsequent microbial growth, and promote the degradation of petroleum hydrocarbons. However, mineral fertilizers are costlier and less convenient than most organic fertilizers. Table 3 summarizes Cases of organic fertilizer remediation.

**Table 3:** Cases of organic fertilizer remediation.

Serial number	Type of pollutant	TPH concentration (mg/kg)	Added substance	Processing cycle	Maximum TPH removal rate	Reference
1	Crude oil	15,000	Soybean straw	120 d	74.06%	Shi et al. (2022)
2	Petroleum	2,278	Saddy straw mineral fertilizers	5 m	45.2%	Huang et al. (2019)
3	Petroleum	45,000	plant litters of *Bothriochloa ischaemum* and *Robinia pseudoacacia*	150 d	About 80%	Zhang et al. (2021b)
4	Engine oil	7,500	Horse manure	5 m	77% (PHC)	Robichaud et al. (2019)
5	Crude oil	21,776	Food waste	30 d	49.3% (TPH/n-alkanes)	Xu et al. (2019)
6	Petroleum	26,300	Aged refuse	210 d	63.7%	Chen et al. (2019b)
7	Diesel oil	8,000	Meat bone meal	28 d	About 90%	Liu et al. (2019)
8	Petroleum	15,000	Compost	60 d	35.7%	Zhang et al. (2011)

### Biochar

Biochar is a carbon-dense byproduct created through the pyrolysis of organic materials (Wang et al., 2024; Rassaei, 2025). Biochar is characterized by a well-developed pore structure, a large specific surface area, specific nutrients, and abundant surface functional groups. It is also widely accessible and cost-effective, making it highly efficient as a biostimulant for the remediation of soils contaminated with petroleum hydrocarbons (Dike et al., 2021; Li et al., 2022a; Zhang et al., 2025).

#### Biochar remediation alone

**Figure 1:**
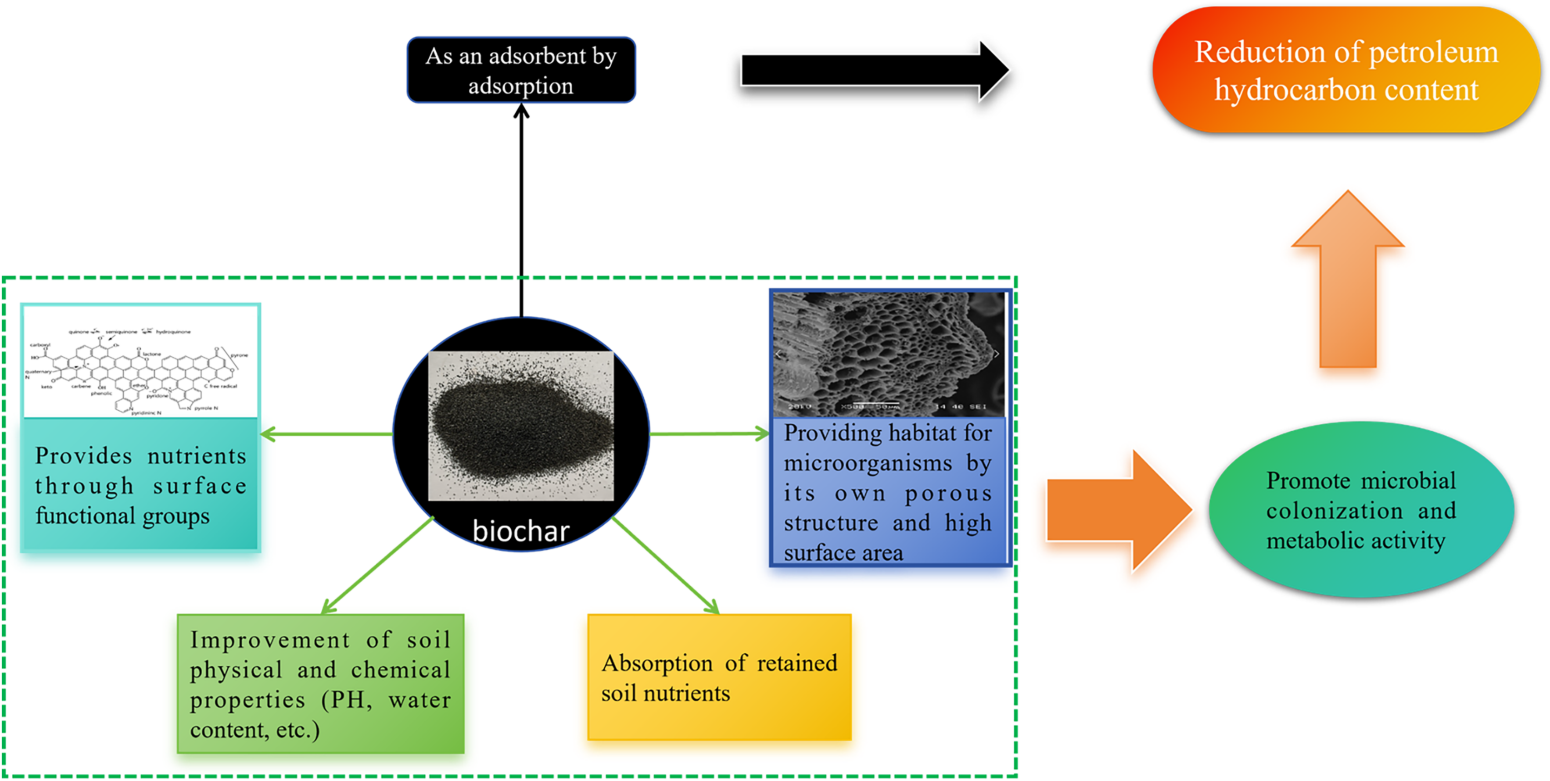
Role of biochar in restoration of petroleum hydrocarbon-contaminated soils.

To begin with, biochar possesses a significant ability to adsorb substances and exhibits enhanced stability, which can efficiently decrease the levels of heavy metals, petroleum hydrocarbons, and various other contaminants (Ali et al., 2019; Mansoor et al., 2021). Biochar primarily enhances the breakdown of petroleum hydrocarbons by stimulating biological activity. It creates an optimal environment for microorganisms, encouraging the proliferation of native microbial populations, which in turn boosts the rate of TPH degradation (Ren et al., 2022; Kong et al., 2018; Li et al., 2022b). Yu et al. (2019) remediated petroleum hydrocarbon contaminated soils by using biochar and mineral fertilizer, and the comparison of them found that their dominant bacterial flora were different, which proved that biochar could promote the certain microbial growth. Second, biochar can improve the water content of soil, which is favorable for the microbial degradation of petroleum hydrocarbons (Saeed et al., 2021; Lee et al., 2022b; Zhang et al., 2019). In addition, biochar materials have surface functional groups that can supplement specific nutrients to soil microorganisms, thereby promoting their growth (Dike et al., 2021; Zama et al., 2018). Saeed et al. (2021) found that the nitrogen content of biochar-treated soils increased by 23% and 16%, phosphorus content by 10–5%, and potassium content by 5–3%, respectively, under 10% and 15% oil contamination compared to the non-biochar treatment. The role of biochar in restoring petroleum hydrocarbon-contaminated soils is illustrated in Fig. 1, highlighting mechanisms such as sorption, microbial stimulation, and reduction of contaminant mobility. Biochar can act through two distinct mechanisms: sorption and nutrient amendment. Sorption refers to biochar’s ability to physically adsorb or chemically bind petroleum hydrocarbons onto its porous surface, reducing their mobility and toxicity. In contrast, nutrient amendment involves biochar supplying essential nutrients (such as nitrogen or phosphorus) to the soil, thereby stimulating microbial activity and enhancing biodegradation. These two mechanisms work independently but can also synergize during remediation.

#### Combined remediation of biochar and surfactants

Biochar is rarely used alone in remediation; it often functions more effectively when paired with other amendments such as surfactants, mineral fertilizers, or organic compost. Biochar serves as an ally for other biostimulants during the process of soil remediation from petroleum hydrocarbon contamination. Bioremediation uses surfactants as one of the most widespread biostimulant groups. Surfactants provide dual lipophilic and oleophilic properties and reduce surface tension to increase the availability of contaminants (Rong et al., 2021; Karlapudi et al., 2018). The application of surfactants generates several negative side effects that affects how effectively petroleum hydrocarbons break down (Sydow et al., 2018). Such negative impacts can be reduced when biochar works together with surfactants as part of a synergistic remediation strategy according to Wei et al. (2020b).

According to Zhen et al. (2021), biochar modification with rhamnolipid decreased biochar surface area and elevated oxygen content which led to improved petroleum hydrocarbon degradation capacity in soil with modified biochar sustaining 32.9% TPH reduction after 90 days while rhamnolipid and biochar co-treatment showed lower degradation performance. The incorporation of rhamnolipids with biochar enables better microbial interaction along with stronger ability to decompose petroleum hydrocarbons from soil.

#### Combined remediation of biochar and mineral fertilizers

Beyond surfactants, mineral fertilizers are another category that shows synergistic potential with biochar. Soils with organic contamination cannot be directly remediated by biochar due to insufficient biochar remediation ability and the soil’s unfavorable carbon-to-nitrogen ratio balance (Wu et al., 2017; Shamloo, Ebrahimi & Charati, 2025). According to Kong, Liu & Wu (2023), the application of biochar together with urea has proven effective for petroleum hydrocarbon polluted soil remediation. The combined method removed 78.6% of total petroleum hydrocarbons from the soil after 80 days of application. Soil pH and total nitrogen along with ammonium ions and microbial population received enhancement when biochar was combined with nitrogen fertilizer while this mixture also elevated dehydrogenase and catalase activity and alkB and CYP gene abundance relative to the control.

The trio of biochar, surfactants and mineral fertilizers can also be combined for remediation. Wei et al. (2020a) used a combination of biochar, rhamnolipid (RL) biosurfactant and nitrogen (N). The results showed that after 50 d, biochar + RL, biochar + N, and biochar + N + RL lowered the total soil pH by 32.3%, 73.2%, and 80.9%, respectively. It showed a synergistic effect and was more effective than individual applications, and the composite treatment significantly promoted the adsorption of aromatic compounds, while RL and N promoted the degradation of heavy and light aliphatic compounds.

#### Combined biochar and compost remediation

In addition to mineral fertilizers, compost can also be co-applied with biochar to enhance microbial activity and organic matter turnover. Biochar features a porous architecture, a significant specific surface area, and numerous surface functional groups, all of which can serve as a habitat and supply nutrients for microorganisms involved in composting. Incorporating biochar into the composting process shortens the composting duration, enhances the breakdown of organic materials and the formation of humus, curtails greenhouse gas emissions, boosts microbial populations, and adjusts the structure of the microbial community (Pang et al., 2023; Zhou et al., 2019). Moreover, the use of biochar has a notable impact on the moisture levels, available oxygen, temperature, pH, and carbon-to-nitrogen ratio during the composting of organic waste, thereby enhancing microbial proliferation (Nguyen et al., 2022).

According to Lv et al. (2022) petroleum hydrocarbon contaminated soil received treatment with rice husk biochar and aerobic composting which resulted in heavy oil degradation to 74.82% during 26 days. Results demonstrated that adding rice husk biochar impact microbial community development throughout composting thus accelerating heavy oil decomposition in the compost process. Biochar holds strong abilities to adsorb to surfaces and precipitate chemicals and possess high ash amounts yet compost (SMC) functions through organic matter content and microbial communities with enzyme activities. The characteristics of rice husk biochar allow better interaction with hydrocarbons and pollutants which decreases their bioavailability and NaCl ion availability. The combined toxic effects on microbial communities and nutrient deficiency become less severe because of this process which results in efficient oil contamination treatment for soil (Bao et al., 2020). The main mechanism through which TPH removal functions in biochar-assisted composting relies on biostimulation instead of adsorption (Lv et al., 2023b). Uyizeye, Thiet & Knorr (2019) reported that combined compost and biochar remediation reached 62% TPH degradation during 90 days which outpaced single biochar and compost remediation results. PAH remediation in the treatment remained ineffective due to the combination of biochar with compost thereby restricting bacterial activities and PAH-degrading functional genes within soil (Bao et al., 2020). Overall, biochar demonstrates the highest remediation potential when used in combination with surfactants, fertilizers, or compost. These integrated strategies enhance nutrient delivery, microbial activation, and hydrocarbon breakdown.

Owing to its unique structure and chemical composition, biochar can synergize with other remediation methods to improve the remediation efficiency. As mentioned earlier, it can synergize well with surfactants, mineral fertilizers, and organic compost. Therefore, in the future, the use of biochar should not be limited to individual remediation, but should focus more on the synergistic effect of other types of remediation agents and research on the remediation effect, Table 4 summarizes the instances of remediation using biochar-based methods.

**Table 4:** Instances of remediation using biochar-based methods.

Serial number	Type of pollutant	TPH concentration (mg/kg)	Added substance	Processing cycle	Maximum TPH removal rate	Reference
1	Crude oil	15,000	Australian pines biochar	40 d	About 23.6%	Saeed et al. (2021)
2	Heavy oil	5,000	Fresh sisal leaves biochar aerogel	60 d	86.62%	Lv et al. (2023a)
3	Crude oil	50,000	Sludge biochar	90 d	32.9%	Zhen et al. (2021)
			Rhamnolipid-modified sludge biochar		About 28%	
4	Petroleum	4,000	Corn cob biochar	80 d	58.6%	Kong, Liu & Wu (2023)
			Corn cob biochar + Urea		78.6%	
5	Petroleum	500	Sugarcane residue biochar	50 d	40.6%	Wei et al. (2020a)
			Biochar + Rhamnolipid		32.3%	
			Biochar + Urea		73.2%	
			Biochar + Urea + Rhamnolipid		80.9%	
6	Heavy oil	2,000	Rice husk biochar + Compost	26 d	74.8%	Lv et al. (2022)
7	Diesel oil	95,333	Biochar	90 d	52.8%	Uyizeye, Thiet & Knorr (2019)
			Biochar + Compost		60.1%	

### Plants

Phytoremediation of petroleum hydrocarbon-contaminated soils is also a very promising remediation technique. Phytoremediation involves many processes for removing pollutants, such as phytovolatilization, phytotransformation, and phytostabilization, all of which involve plants and their microbiota, but rhizoremediation is the main process of phytoremediation (Cheng, Zhou & Yu, 2019; Correa-García et al., 2018). Cheng, Zhou & Yu (2019) found that phytoremediation degraded PHCs in soil by as much as 37.6–53.3%, but the proportion of which phytoaccumulation accounted for a low percentage of 0.3–13.3%, indicating that rhizoremediation contributed the most to the removal of PHCs. Rhizoremediation is a biostimulation method for the remediation of TPH-contaminated soils, which focuses on the removal of organic pollutants by stimulating the metabolic activity of rhizosphere microbiome through plant synergism (Hussain et al., 2018; Sattar et al., 2022). The microbial community is an important component of plant interroots and plays a major role in the rhizoremediation of petroleum pollutants (Kotoky, Rajkumari & Pandey, 2018). Plant root exudates can stimulate the metabolic activity of rhizosphere microbiome and play a key role in enhancing TPH biodegradation (Kotoky, Rajkumari & Pandey, 2018; Chen et al., 2019a; Omenna et al., 2024). Therefore, rhizoremediation plants act as stimulants for microorganisms by providing a suitable environment and stimulating them through root exudates to remediate petroleum hydrocarbon-contaminated soils. Rhizoremediation occurs when plant roots interact with soil microbes in the rhizosphere. Roots exude organic compounds that serve as carbon sources for petroleum-degrading bacteria. Additionally, root systems increase oxygen availability and improve soil structure, both of which enhance microbial activity and hydrocarbon degradation.

#### Restoration of plants alone

In rhizoremediation and phytoremediation, the chemical toxicity of pollutants is not the only factor influencing plant growth and development; physical impacts like decreased oxygen levels and poor soil structure can also impact the effectiveness of phytoremediation (Hussain et al., 2019; Nwajiobi et al., 2025). Therefore, it is important to select suitable plants for the remediation of petroleum hydrocarbon-contaminated soils. For example, preference should be given to the use of native plants that are tolerant to petroleum hydrocarbons over exotic plants, as this will provide an economically viable and environmentally sustainable option for reclaiming petroleum hydrocarbon-contaminated sites at any given location (Correa-García et al., 2018; Hoang et al., 2021b; Singha & Pandey, 2021). Camacho-Montealegre, Rodrigues & Tótola (2019) tested 12 plants *Sorghum arundinaceum* Desv, *Oryza longistaminata* A., Hy- parrhenia rufa Nees, *Abelmoschus ficulneus* L. and other native plants for remediation of petroleum hydrocarbon contaminated soils and found that *H. rufa* (Gramineae) had the highest degradation of petroleum hydrocarbons of 74.4% after 120 d and had the highest potential for petroleum tolerance. The pristine environment plants themselves in some petroleum contaminated areas are sufficient to remediate petroleum hydrocarbon contamination without human intervention. Meištininkas et al. (2023) found that the pristine environment of an island had a range of 66% to 75% biodegradation of total petroleum hydrocarbons after 60 days of aerobic conditions, which proves that the rhizosphere microbiome in pristine soils have the ability to eliminate hydrocarbons through biodegradation. In rhizoremediation and phytoremediation, both chemical toxicity and physical constraints such as poor soil structure and low oxygen availability can hinder plant growth and remediation effectiveness (Hussain et al., 2019; Nwajiobi et al., 2025). Selecting appropriate plant species is crucial. Native, petroleum-tolerant species are generally preferred over exotic plants, as they offer cost-effective and environmentally sustainable solutions for remediating contaminated sites.

(1) The nitrogen fixation capabilities of specific legumes make them optimum for TPH-contaminated soil remediation which produces better results compared to all other non-legume plants. The trials of Meištininkas et al. (2023) demonstrated that *M. sativa* and *L. corniculatus* together with *M. albus* displayed the highest petroleum oil removal performance and survival tolerance reaching 95% after 90 days of remediation. *M. albus* (MA) had the best restoration of soil nutrients (nitrogen and phosphorus) (Allamin et al., 2020). The best performance for remediation of soil nutrients was achieved by *L. corniculatus* (LC) combined with *M. albus* (MA). The combined ability of *M. albus* (MA) brought restoring soil nutrients to their best possible levels with phosphorus and nitrogen. Studies conducted by Farouq et al. (2022) showed *Vigna unguiculata* root exudates establish major soil alterations which boost both microbial community quantities and functionality. Xu et al. (2021) evaluated the remediation performance of five different plants Susanna, Seep weeds, and Sea-lavender for petroleum hydrocarbon treatment in contaminated soils. Results demonstrated Sesbania produces large amounts of biomass and exhibits superior petroleum hydrocarbon degradation capacity than other studied plants thus qualifying it as an excellent choice for coastal wetlands hydrocarbon-contaminated soil remediation.
(2) Scientists have documented how Poaceae plants deliver persuasive pollution removal capabilities (Olson et al., 2007; Dong et al., 2019). The high plasticity together with environmental adaptability of Poaceae enables these plants to work effectively at cleaning petroleum hydrocarbon contaminated soils (Guarino et al., 2020). Tall fescue, a perennial poaceae, is widely used for rhizoremediation because of its tolerance to pollutants and its fibrous root system (Dong et al., 2019; Steliga & Kluk, 2020). Lee et al. (2021) demonstrated the remediation potential of tall fescue by finding that the TPH removal rate of tall fescue for 37 d was 30.2%, which was significantly higher than that of unplanted soil at 19.4%. Lee et al. (2022a) investigated tall fescue’s 317 d long-term remediation of petroleum hydrocarbon contaminated soil capacity, the results showed that the maximum remediation rate of 75.6% occurred in 61 d, and it was found that the structure of its rhizosphere microbiome changed with the season, and the degradation rate of TPH was positively proportional to that of the petroleum hydrocarbon degrading bacteria, such as Lysobacter. The Poaceae *Cynodon* spp. and *Agropyron desertorum* proved to be effective agents for phytoremediation (TPH degradation rates of 38.9% and 30.4% respectively) based on findings reported by Saraeian et al. (2018). Two varieties of the Poaceae family named *Brachiaria decumbens* and *Megathyrsus maximus* prove suitable for petroleum hydrocarbon-contaminated soil remediation while enhancing soil fertility levels (Uribe, Peñuela & Pino, 2022). Ma et al. (2023) investigated how different sweet sorghum varieties performed at remediation of petroleum hydrocarbons from petroleum-contaminated saline soils achieving maximum remediation rate at 69.3%. The remediation potential of additional plant families besides the described two still needs to be explored for petroleum hydrocarbon-contaminated soils; for example, plants in the family of *Sanguisorba* are also able to effectively remediate petroleum hydrocarbon-contaminated soils (Hoang et al., 2021a).

**Figure 2:**
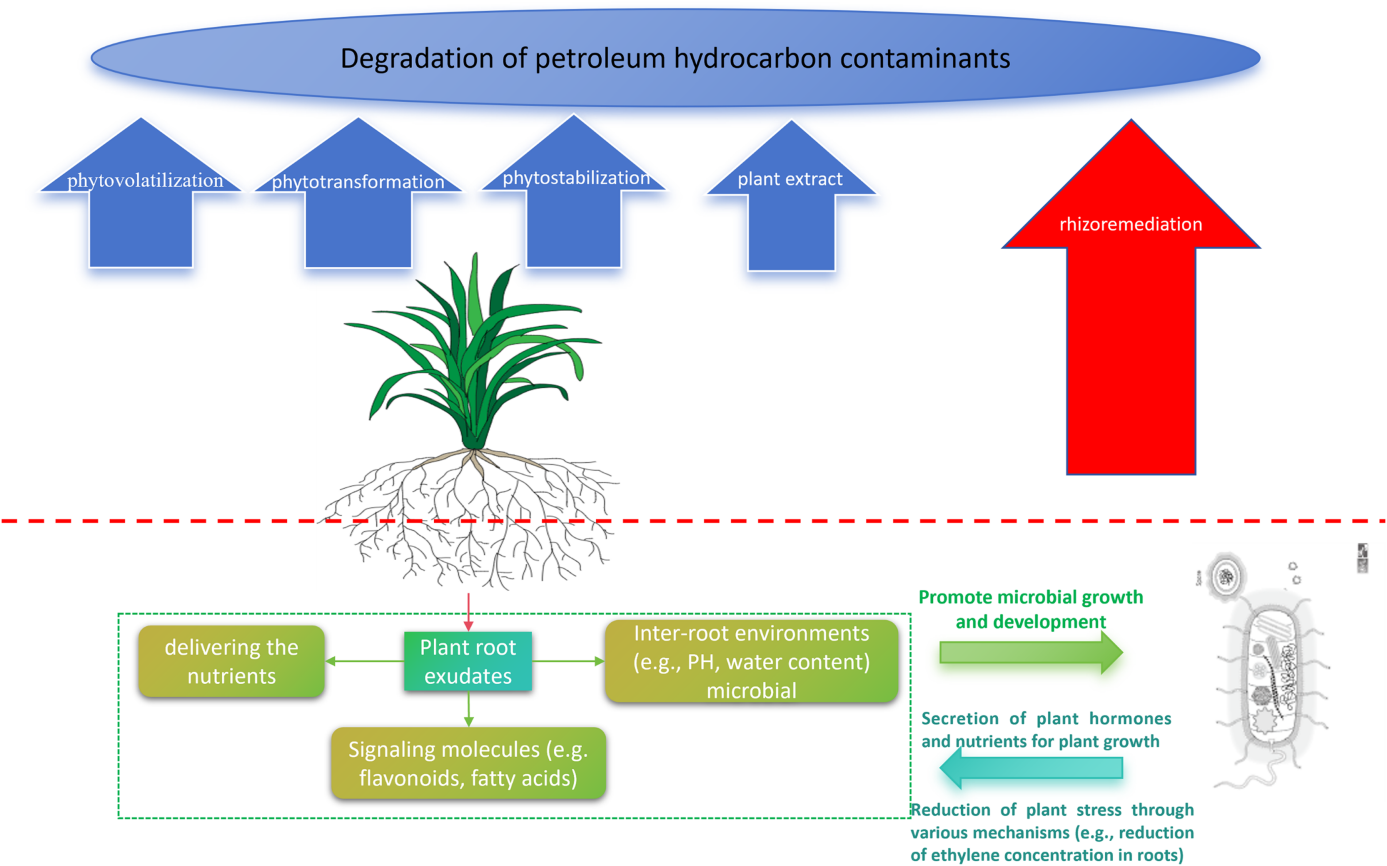
Primary functions of phytoremediation.

The selection of suitable phytoremediation should be based on local conditions, that is, it is best to utilize local plant species to remediate local contaminated sites, which is conducive to the adaptation of plants to the environment, thus improving remediation efficiency. The sustainability of phytoremediation is strong because of the mutually beneficial symbiotic relationship between plant rhizoremediation and petroleum hydrocarbon-degrading microorganisms. However, compared to other biostimulants, the short-term degradation rate of petroleum hydrocarbons may be lower; therefore, a longer restoration period is required. Currently, most plant species that are suitable for degrading petroleum hydrocarbons are from the Poaceae and legume families, and there is a need to explore plant species with high degradation efficiency in the future. The primary mechanisms of phytoremediation, including phytoextraction, phytodegradation, and phytostabilization, are summarized in Fig. 2 to demonstrate plant-based remediation functions.

#### Combined plant and organic fertilizer restoration

Phytoremediation alone is often limited and influenced by environmental conditions, so adding other biostimulants to phytoremediation can lead to better remediation of petroleum hydrocarbon contaminated soils (Li et al., 2023b). Rhizoremediation is a synergistic form of bioremediation and phytoremediation that improves the overall remediation performance and remediation rate through the synergistic action of plant roots and microorganisms. The overall performance and efficiency of rhizoremediation can be improved by selecting appropriate plant and microbial pairings and adding soil amendments, such as organic and inorganic amendments (Hussain et al., 2018).

Addition of organic fertilizers to rhizoremediation can provide microorganisms with nutrients and other elements, thus improving the remediation efficiency. Ruley et al. (2020) used organic fertilizer cow dung in combined remediation with plants, and the results demonstrated that the application of cow dung combined with an effective phytoremediation agent significantly increased the reduction of TPH as compared to natural attenuation or the use of manure or phytoremediation agent alone. Seo & Cho (2020), Wang et al. (2021a) added organic compost to phytoremediation, and the results showed that organic compost treatment resulted in a decrease in soil pH, an increase in the removal of TPHs, alkanes, and aromatics, an increase in the activities of soil dehydrogenase and PPOs enhanced, and a relative increase in PAHs and alkane-degrading bacteria. In addition, the organic fertilizer significantly increased aboveground length, root vigor, and aboveground and root dry weights of *Vetiveria zizanioides*. The targeted addition of mineral fertilizers (N, P) to plant rhizoremediation can also significantly improve the removal efficiency of TPH (Hoang et al., 2022).

#### Combined plant and exogenous bioremediation

Additional factors that determine the success of TPH remediation through rhizoremediation include how well plants tolerate TPH and the capability of the degrading microorganisms to occupy developing root systems and their ability to break down TPH compounds in the first place. The success of TPH remediation becomes more efficient when suitable plant-microbe partnerships are developed. Petroleum hydrocarbon-degrading bacteria isolated from petroleum-contaminated areas can be utilized for combined remediation with plants. Promsinga et al. (2021) utilized petroleum hydrocarbon-degrading bacteria screened from petroleum-contaminated soils with vetiver and the results showed that *Vetiveria zizanioides* and the bacteria *M. luteus* WN 01 or Kocuria sp. MU01 had a stronger remediation of petroleum hydrocarbon-contaminated soils and that after 45 d. The combination of vetiver and *M. luteus* WN01 showed the best TPH degradation after 45 d (TPH degradation rate of 50.25%). Eze et al. (2022) demonstrated that petroleum-degrading bacteria isolated from petroleum-contaminated sites can effectively increase the biomass of plants, and the combination of these with *M.sativa* resulted in a TPH degradation rate of 91% after 60 d. The incorporation of petroleum hydrocarbon-degrading bacteria leads to better TPH breakdown alongside lower toxicity against plant life and supports better plant growth development (Ali et al., 2023; Auti et al., 2019). Therefore, the combination of microorganisms isolated from inter-roots of plants and plants can also effectively improve the TPH degradation rate (Song et al., 2021; Afegbua & Batty, 2019). Lee, Lee & Cho (2023) found that Novosphingobium sp. CuT1, isolated from the inter-roots of tall fescue, can effectively promote plant growth and TPH degradation (Afegbua & Batty, 2019). Ani et al. (2021) also demonstrated that the combination of inoculated rhizoremediation microorganisms and plants was much more effective in TPH degradation than isolated microorganisms and plants alone.

Based on combined bioaugmentation and phytoremediation, the addition of mineral fertilizers (N, P, K, *etc*.) to stimulate microorganisms can achieve improved TPH degradation (Shahzad et al., 2020). Bhuyan, Kotoky & Pandey (2023) added NPK for bioaugmentation based on plant inoculation with rhizoremediation-isolated bacteria. The results demonstrated that when bacterial strains were used with NPK biostimulant, they could improve plant growth and attenuated plant and soil enzyme activities at petroleum contaminated sites, and bacterial aggregation-NPK biostimulation also altered the rhizoremediation microbiome and increased TPH degradation in crude oil contaminated soils. Ubogu, Odokuma & Akponah (2019) has also demonstrated that combined remediation of mineral fertilizers and exogenous petroleum hydrocarbon-degrading bacteria with plants is more effective than biostimulation or bioaugmentation alone. Anwar-ul-Haq et al. (2022) employed a combination of surfactants, exogenous microorganisms, and phytoremediation, thus proving that this combined application can help in the restoration of petroleum-contaminated soils for agricultural use.

#### Combined plant and biochar remediation

Plant root exudates are beneficial to biochar, and coapplication of biochar with root secretions can address the reduced bioavailability of hydrocarbons associated with biochar research (Ni et al., 2018; Dike et al., 2022). Biochar and inter-root soils were found to be enriched with some PAH-degrading bacteria, and the combination of these two promoted synergistic effects among PAH-degrading bacteria (Li et al., 2020). Therefore, the combined application of plants and biochar amendments is beneficial for the remediation of contaminated soil. Yousaf et al. (2022) showed that the TPH removal rate of the combined remediation of biochar and plants was higher than that of plants alone as well as biochar remediation, which proved that biochar promoted phytoremediation. In addition, the addition of biochar to phytoremediation not only promotes rhizoremediation to increase the TPH degradation rate but also promotes plant growth and development (Hussain et al., 2022). Combined phytoremediation strategies consistently outperform plant-only remediation by enhancing microbial activity, nutrient cycling, and contaminant bioavailability. Organic fertilizers such as compost and cow dung not only improve plant growth but also stimulate microbial degradation of TPHs. The addition of biochar enhances soil physicochemical properties and supports rhizobacterial colonization. Inoculation with petroleum-degrading microbes further accelerates biodegradation. Overall, the synergistic use of plants with biostimulants whether organic amendments, exogenous microbes, or biochar leads to faster, more complete remediation. Future efforts should focus on tailoring plant microbe amendment combinations based on site-specific conditions to optimize efficiency and sustainability.

Adopting the joint restoration of plants and other biostimulants can compensate for the shortcomings of plant restoration alone, improve restoration efficiency, and shorten the restoration cycle. Adding other biostimulants to phytoremediation can also improve the restoration environment for plants. For example, adding fertilizers can provide nutrients for plants, and adding biochar contributes to the enhancement of soil physicochemical properties., which is conducive to phytoremediation. The sustainability of phytoremediation is strong, but the efficiency of plants alone is not high. Adding other biostimulants to phytoremediation can improve the remediation efficiency, so the main trend in the future is to use phytoremediation in combination with other biostimulants.

### Summary of biostimulant applications in TPH-contaminated soils

To consolidate the findings from recent studies on biostimulant efficacy, Table 5 presents a comparative overview of commonly used materials, soil conditions, pollutant types, treatment durations, microbial impacts, and achieved TPH removal rates. This table highlights the variability in performance based on material type and soil environment, offering insight into optimized biostimulant applications for petroleum hydrocarbon remediation.

**Table 5:** Phytoremediation and integrated phytoremediation approaches.

Serial number	Type of pollutant	TPH concentration	Added substance	Processing cycle	Maximum TPH removal rate	Reference
1	Heavy oil	2,500 mg/kg	*M. sativa* (Legume)	90 d	94.80%	Meištininkas et al. (2023)
2	Petroleum	1%	*Cajanus cajan* (Legume)	90 d	92%	Allamin et al. (2020)
3	Diesel oil	9,064 mg/kg	Tall fescue (Poaceae)	85 d	67.2%	Lee et al. (2021)
4	Diesel oil	30,000 mg/kg	Tall fescue (Poaceae)	61 d	75.6%	Lee et al. (2022a)
5	Petroleum	38,750 mg/kg	Cynodon spp. (Poaceae)	7 m	51.2%	Saraeian et al. (2018)
			*Agropyron desertorum* (Poaceae)		47.4%	
6	Heavy oil	75,000 mg/Kg	*Hyparrhenia rufa* (Poaceae)	120 d	About 73%	Ruley et al. (2020)
			*Hyparrhenia rufa* + Cow dung		About 92%	
7	Petroleum	4%	*Lolium multiflorum* (Poaceae)	75 d	59.55%	Yousaf et al. (2022)
			*Lolium multiflorum* + Compost		68.5%	
			*Trifolium repens* (Legume)		27.25%	
			*Trifolium repens* + Biochar		68%	

To consolidate the diversity of biostimulant strategies reviewed in the previous sections, Tables 6 and 7 presents a comparative overview of recent studies. It covers biostimulant types, target pollutants, treatment durations, microbial responses, and total petroleum hydrocarbon (TPH) removal efficiencies. This comparative summary provides insight into which combinations are most effective and under what conditions.

**Table 6:** Comparative summary of biostimulant materials, treatment conditions, and TPH removal efficiencies in contaminated soils (1–10).

S/N	1	2	3	4	5	6	7	8	9	10
Biostimulant type	Biochar	Plant-based	Biochar	Plant-based	Agricultural	Plant-based	Agricultural	Agricultural	Domestic waste	Mineral fertilizer
Material	Food waste	Poultry manure	KNO_3_	Food waste	Poultry manure	Corn cob biochar	Sludge biochar	KNO_3_	Tall fescue	Meat bone meal
Pollutant	Petroleum	Diesel	Diesel	Petroleum	Crude oil	Diesel	Petroleum	Crude oil	Crude oil	Petroleum
TPH (mg/kg)	40,959	27,929	34,641	38,291	5,878	7,526	45,657	5,126	25,017	46,502
Soil type	Sandy loam	Clay	Loamy	Sandy loam	Peaty	Silt loam	Loamy	Sandy loam	Loamy	Clay
Soil pH	7.5	6.9	6.5	7.9	6	6.9	6.9	7.7	7.4	7.6
Moisture (%)	26	25	19	32	28	35	30	32	35	28
Temp (°C)	35	21	30	31	25	24	23	25	31	21
Duration (days)	143	54	57	31	60	146	132	127	37	68
C/N ratio	100.10.00	120.10.00	80.10.00	80.10.00	80.10.00	100.20.00	100.20.00	120.10.00	120.10.00	100.20.00
P content	Low	Low	Low	High	Medium	High	Low	Medium	Low	Low
K content	Moderate	Low	Low	Moderate	Moderate	High	Low	Moderate	Moderate	Low
Application method	Intermittent	Drip	Pulse-fed	Batch-fed	Pulse-fed	Intermittent	Drip	Batch-fed	Drip	Pulse-fed
Microbial response	â†‘ Activity	â†‘ Diversity	â†‘ Activity	â†‘ Activity	â†‘ Diversity	â†‘ Abundance	â†‘ Abundance	â†‘ Activity	â†‘ Diversity	â†‘ Abundance
TPH removal (%)	60	89	81	41	89	59	77	52	77	88
Additional agent	Surfactant	Cow Dung	Cow Dung	Biochar	Biochar	Compost	Surfactant	None	None	Compost
Soil improvement	â†‘ Porosity	â†‘ Fertility	Neutral	Neutral	Neutral	Neutral	Neutral	â†‘ Fertility	â†‘ Fertility	Neutral
Experimental scale	Lab-scale	Field-scale	Lab-scale	Lab-scale	Field-scale	Lab-scale	Field-scale	Lab-scale	Lab-scale	Lab-scale
Synergy observed	Yes	No	No	Yes	Yes	No	No	No	No	No
Degradation mechanism	Oxidation	Enzymatic breakdown	Adsorption & Biodegradation	Adsorption & Biodegradation	Oxidation	Enzymatic breakdown	Enzymatic breakdown	Enzymatic breakdown	Adsorption & Biodegradation	Oxidation
Nutrient strategy	Intermittent	Intermittent	Drip	Pulse-fed	Pulse-fed	Drip	Batch-fed	Drip	Pulse-fed	Pulse-fed
Monitoring method	qPCR	16S rRNA	GC-MS	Metagenomics	qPCR	Metagenomics	GC-MS	16S rRNA	Metagenomics	qPCR
Reference	Zhang et al. (2021b)	Ruley et al. (2020)	Liu et al. (2019)	Zhang et al. (2021b)	Ruley et al. (2020)	Liu et al. (2019)	Dike et al. (2024)	Liu et al. (2019)	Lee et al. (2021)	Liu et al. (2022)

**Table 7:** Comparative summary of biostimulant materials, treatment conditions, and TPH removal efficiencies in contaminated soils (11–20).

S/N	11	12	13	14	15	16	17	18	19	20
Biostimulant type	Domestic waste	Plant-based	Mineral fertilizer	Plant-based	Agricultural	Domestic waste	Mineral fertilizer	Mineral fertilizer	Agricultural	Agricultural
Material	Sludge biochar	Soybean straw	Corn cob biochar	Tall fescue	Food waste	Sludge biochar	Tall fescue	Meat bone meal	Poultry manure	KNO_3_
Pollutant	Petroleum	Diesel	Petroleum	Petroleum	Petroleum	Crude oil	Crude oil	Petroleum	Diesel	Diesel
TPH (mg/kg)	19,968	49,326	42,241	23,720	10,453	45,606	46,088	29,765	46,245	36,937
Soil type	Loamy	Loamy	Loamy	Loamy	Sandy loam	Silt loam	Loamy	Sandy loam	Silt loam	Sandy loam
Soil pH	6.6	8.2	7	7.1	7.5	6.6	6.3	8.2	7.7	6.2
Moisture (%)	20	25	35	28	26	19	19	15	15	24
Temp (°C)	26	21	22	27	34	25	28	23	32	22
Duration (days)	116	111	104	71	128	85	38	31	96	117
C/N ratio	100.10.00	80.10.00	120.10.00	100.20.00	100.10.00	100.05.00	80.10.00	100.20.00	100.10.00	100.05.00
P content	Low	High	Low	Low	Medium	Low	Medium	High	High	Medium
K content	High	High	Low	High	Moderate	High	High	Moderate	Moderate	Moderate
Application method	Batch-fed	Batch-fed	Intermittent	Drip	Intermittent	Batch-fed	Drip	Intermittent	Intermittent	Drip
Microbial response	â†‘ Activity	â†‘ Abundance	â†‘ Activity	â†‘ Abundance	â†‘ Abundance	â†‘ Abundance	â†‘ Abundance	â†‘ Abundance	â†‘ Activity	â†‘ Activity
TPH removal (%)	57	49	43	45	87	80	54	80	41	43
Additional agent	Biochar	None	None	Surfactant	Compost	Biochar	Biochar	Surfactant	Compost	Surfactant
Soil improvement	â†‘ Fertility	â†‘ Porosity	â†‘ Porosity	â†‘ Fertility	Neutral	â†‘ Moisture	â†‘ Porosity	â†‘ Fertility	Neutral	â†‘ Moisture
Experimental scale	Lab-scale	Lab-scale	Lab-scale	Field-scale	Lab-scale	Lab-scale	Lab-scale	Field-scale	Lab-scale	Lab-scale
Synergy observed	No	Yes	No	No	Yes	No	No	Yes	No	Yes
Degradation mechanism	Oxidation	Adsorption & Biodegradation	Oxidation	Enzymatic breakdown	Adsorption & Biodegradation	Adsorption & Biodegradation	Adsorption & Biodegradation	Adsorption & Biodegradation	Adsorption & Biodegradation	Adsorption & Biodegradation
Nutrient strategy	Intermittent	Drip	Drip	Batch-fed	Intermittent	Intermittent	Pulse-fed	Pulse-fed	Pulse-fed	Drip
Monitoring method	GC-MS	Metagenomics	qPCR	Metagenomics	qPCR	Metagenomics	16S rRNA	16S rRNA	Metagenomics	Metagenomics
Reference	Dike et al. (2024)	Shi et al. (2022)	Liu et al. (2019)	Lee et al. (2021)	Zhang et al. (2021b)	Dike et al. (2024)	Lee et al. (2021)	Liu et al. (2022)	Ruley et al. (2020)	Liu et al. (2019)

As seen in Tables 6 and 7, biostimulant performance varies depending on the material composition and site-specific soil conditions. Biochar-nitrogen fertilizer combinations consistently yield high removal rates, while organic waste amendments like food waste and manure support microbial activity and enhance biodegradation. This comparative data reinforces the importance of selecting site-appropriate biostimulants and supports the case for combined biostimulant strategies tailored to contamination characteristics. The data presented clearly illustrate that the effectiveness of biostimulation strategies in remediating TPH-contaminated soils is highly dependent on the type of biostimulant material, application method, and site-specific soil conditions. Organic amendments such as food waste and poultry manure demonstrated strong microbial activation and high TPH removal rates under both lab- and field-scale conditions. Mineral fertilizers like KNO_3_ were particularly effective in nitrogen-deficient soils, especially when applied *via* drip or pulse-fed systems. Sludge biochar and compost not only improved microbial abundance but also enhanced soil fertility and porosity. Notably, combinations of biostimulants and synergistic applications especially those incorporating surfactants or co-amendments resulted in enhanced degradation efficiencies. These findings support the growing consensus that site-specific, tailored biostimulation strategies, backed by microbial monitoring and proper nutrient balance, are key to optimizing remediation outcomes in petroleum-contaminated soils.

### Emerging technologies in biostimulation

#### Nanotechnology

The rapid evolution of biotechnological and environmental engineering tools has expanded the potential of biostimulation strategies for remediating petroleum hydrocarbon-contaminated soils. Emerging technologies such as nanotechnology, genetic engineering of microbial strains, and advanced monitoring systems are revolutionizing the way biostimulants are applied and monitored in the field.

Nanotechnology-based interventions have gained momentum due to their ability to enhance bioavailability and degradation kinetics of hydrophobic contaminants. Nanoparticles such as nano-iron oxides, nano-zeolites, and carbon-based nanomaterials (*e.g*., carbon nanotubes, graphene oxide) have demonstrated the capacity to improve nutrient delivery, adsorb hydrocarbons, and provide catalytic surfaces that stimulate microbial activity (Ren et al., 2022; Rong et al., 2021). These materials can be engineered for targeted release and controlled dispersion in subsurface environments, minimizing nutrient losses and enhancing contaminant-microbe interactions.

#### Genetically engineered microbes (GEMs)

Genetically engineered microorganisms (GEMs) offer another frontier for biostimulation enhancement. Through synthetic biology approaches, native strains can be modified to overexpress hydrocarbon-degrading enzymes or stress-tolerant traits, thereby improving their survival and activity in harsh contaminated environments. For example, engineered strains with elevated expression of *alkB* or *CYP450* genes have been shown to significantly accelerate the breakdown of long-chain hydrocarbons. Furthermore, the development of microbial consortia with complementary metabolic capabilities allows for more comprehensive degradation of complex pollutant mixtures (Ren et al., 2022; Bhuyan, Kotoky & Pandey, 2023).

#### AI-based monitoring systems

Concurrently, biosensor-integrated and AI-enhanced monitoring systems are enabling real-time assessment of remediation progress and microbial activity. Biosensors based on microbial reporters or enzyme activity can detect specific hydrocarbon metabolites, allowing *in situ* tracking of degradation dynamics. Additionally, machine learning algorithms applied to omics data (*e.g*., metagenomics, proteomics) and environmental variables can predict system behavior, optimize nutrient ratios, and reduce reliance on trial-and-error strategies. These technologies support precision biostimulation, where interventions are continuously adjusted to match microbial needs and environmental responses (Kong, Liu & Wu, 2023; Anwar-ul-Haq et al., 2022).

Together, these emerging technologies offer substantial improvements in efficiency, adaptability, and environmental control. Integrating nanomaterials, GEMs, and smart monitoring tools into biostimulation strategies represents a paradigm shift in bioremediation, moving toward more predictive, responsive, and site-specific remediation systems.

## Prospects for engineering applications of biostimulation for soil remediation

As a kind of cross-application between environment and geotechnics, there are fewer studies on the engineering properties of biostimulation remediation soil, and this section will be supplemented by the prospect of engineering application from biostimulation remediation soil. The mechanical properties of petroleum hydrocarbon-contaminated soils are more affected by petroleum hydrocarbons than those of ordinary soils. The impact of various hydrocarbon pollutants on the geotechnical characteristics of contaminated soils is influenced by numerous factors, including the type of soil, its gradation and plasticity, the kind of hydrocarbon, the method of sample preparation, and the duration of contamination (Ahmadi, Ebadi & Maknoon, 2021).

High petroleum hydrocarbon contents in soil materials generally create adverse effects on soil mechanical characteristics (Zhang et al., 2021a; Yazdi & Sharifi Teshnizi, 2021). The contamination levels of petroleum hydrocarbons must decrease through the biostimulation method presented in this article to make them suitable for engineering applications (Mir Mohammad Hosseini et al., 2019). The application of thermal desorption methods to soil contaminants produces petroleum hydrocarbon-impacted soils with better engineering properties that have been scientifically proven (Li et al., 2023a). The main benefit of microbial remediation methods arises from the natural occurrence of microorganisms in the soil that improves engineering properties of this material. (Salimnezhad, Soltani-Jigheh & Soorki, 2021). The application of bioremediation methods reduced all measured characteristics of maximum dry unit weight, unconfined compressive strength, swelling index, free swelling and swelling pressure in the evaluated soil material. This remediation increased both soil water content at optimum state along with shear strength while also enhancing cohesive power and internal angle of friction and destructive strain levels and porosity and compression index values. The properties of contaminated soil suffer due to oil contamination yet bioremediation successfully restores those properties to better condition. The documented research findings show potential for biological treatment to cleanse soil materials for engineering applications.

This article examines the scarce research completed to identify engineering uses of biostimulation following soil remediation efforts. Choosing suitable biostimulants remains the essential factor for this method to apply to different contaminated sites. The wide range of biostimulants accompanies distinct mechanical property effects on petroleum hydrocarbon-contaminated soils when implemented with each biostimulant. The research by Narani et al. (2022) investigated petroleum hydrocarbon-contaminated soils treated with organic fertilizer wood chips to demonstrate that wood chips increased concrete shear strength but lowered unconfined compressive strength according to SEM imaging that confirmed woodchip soil remediation and enhancement. Future research on biostimulation applications for soil remediation will center on the usage of organic fertilizers together with biochar-based biostimulants that exhibit their beneficial properties through their solid composition to enhance soil strength properties. This research examines how biostimulants affect soil engineering properties as well as how stimulated indigenous microorganisms influence these properties.

The core mechanism of the biostimulation method is microbial remediation of petroleum hydrocarbons, and a large number of petroleum hydrocarbon-degrading bacteria exist in biostimulated remediated soils, which secrete catalysts, such as urease, which can also be involved in subsequent research on microbial reinforcement. Therefore, future engineering applications for biostimulation soil remediation are promising.

### Engineering applications and policy-relevant considerations of biostimulation

The transition from laboratory or mesocosm-scale experiments to field-scale implementation remains a critical bottleneck in the widespread adoption of biostimulation for remediating petroleum-contaminated soils. Real-world applications must account for diverse variables, including soil heterogeneity, contamination depth, hydrology, climatic conditions, economic feasibility, and long-term sustainability. For instance, a field-scale remediation project in Ogoniland, Nigeria, successfully employed cow dung and poultry manure as biostimulants, achieving over 70% degradation of total petroleum hydrocarbons (TPH) within 90 days under tropical conditions. Similarly, in Alberta, Canada, the combined application of nitrogen and phosphorus fertilizers along with mechanical tilling resulted in a 55% reduction of diesel-range hydrocarbons within 6 months. These case studies exemplify the promise of biostimulation under varied field conditions when appropriately adapted.

A qualitative SWOT analysis (Strengths, Weaknesses, Opportunities, and Threats) provides further insights into the scalability of biostimulation. Its key strengths include eco-friendliness, the ability to enhance indigenous microbial communities, and lower cost compared to physicochemical treatments. However, limitations include slower degradation rates than chemical methods and the need for precise nutrient balancing and real-time monitoring. Biostimulation also presents opportunities for integration with emerging technologies such as biosensors, artificial intelligence, and Internet of Things (IoT) based control systems, which can help optimize remediation strategies in real time. On the other hand, potential threats such as nutrient leaching, inconsistent regulatory frameworks, and concerns related to the use of genetically modified microbes must be carefully addressed. Tackling these challenges through interdisciplinary collaboration and adaptive policy frameworks will be essential for the successful implementation of biostimulant-based remediation systems at the field scale.

#### Field-scale implementation challenges and solutions

At larger scales, the uniform distribution of biostimulants becomes technically complex. Subsurface heterogeneity can lead to uneven amendment delivery, nutrient leaching, or local microbial inhibition. To overcome this, precision delivery systems such as drip irrigation, deep soil injection, or controlled-release nutrient capsules have been developed to optimize amendment dispersion while reducing operational costs and nutrient loss (Wu et al., 2020). Additionally, *in situ* aeration techniques or passive oxygenation through biochar incorporation can help maintain oxygen levels crucial for aerobic hydrocarbon degradation.

Economic viability is another key consideration. Organic waste-based biostimulants like compost, manure, or food waste slurry are typically low-cost and locally available, making them suitable for large-scale remediation in resource-limited settings. In contrast, engineered solutions (*e.g*., nano-fertilizers or biosensors) may involve higher upfront costs but offer greater efficiency and monitoring precision. Thus, cost-benefit analysis should guide the selection of biostimulation strategies based on project scale, urgency, and regulatory constraints.

#### Risk management and regulatory alignment

Despite its sustainability, biostimulation can introduce environmental risks if poorly managed. Over-application of nutrients may lead to eutrophication of nearby water bodies or alter soil microbial equilibrium. To mitigate such risks, field applications should incorporate real-time monitoring of nutrient levels, microbial activity, and degradation kinetics using biosensors or periodic soil sampling. Risk assessments should also evaluate potential by-products of incomplete degradation (*e.g*., polycyclic aromatic hydrocarbons or intermediate metabolites).

On the policy front, regulatory guidance remains limited for biostimulation in many regions. There is a growing need for the development of standardized field protocols and approval pathways for biostimulant materials, particularly for those derived from waste streams or involving microbial inoculants. Establishing clear thresholds for TPH reduction, post-remediation soil quality, and ecological restoration benchmarks will support both public acceptance and environmental compliance.

#### Industry and stakeholder engagement

Industry professionals and environmental managers require practical guidance on the operationalization of biostimulation. This includes **best practices for site assessment**, biostimulant selection based on soil chemistry and contaminant profile, and timelines for expected remediation outcomes. Case studies demonstrating successful field-scale projects, particularly under regulatory oversight or public-private partnerships, could foster wider adoption. Stakeholder engagement—including local communities, environmental agencies, and waste management sectors—can further enhance the feasibility and accountability of field applications.

In summary, engineering-scale applications of biostimulation demand interdisciplinary coordination, technology transfer from lab to field, and policy frameworks that balance environmental efficacy with economic and regulatory realities. Addressing these challenges will be key to unlocking the full potential of biostimulation in restoring petroleum-contaminated soils at a national and global scale.

### Spatial and regional variability in biostimulation outcomes

The performance of biostimulation-based remediation is highly sensitive to geographic and regional variables, including climate, soil physicochemical characteristics, vegetation cover, and the origin and composition of petroleum contamination. These factors can significantly influence microbial activity, nutrient dynamics, contaminant bioavailability, and overall treatment outcomes.

Climatic conditions such as temperature and rainfall play a pivotal role in shaping microbial metabolic rates and moisture-dependent diffusion of nutrients and hydrocarbons. In tropical and subtropical regions, higher soil temperatures and humidity can enhance biodegradation kinetics but may also accelerate nutrient loss through volatilization or leaching. Conversely, in temperate or arid regions, lower temperatures may suppress microbial activity and slow TPH degradation, necessitating longer treatment durations or supplemental aeration.

Soil texture and structure also govern the efficacy of biostimulation. Sandy soils, while promoting aeration, often lack sufficient organic matter and nutrient retention, leading to lower microbial biomass and rapid nutrient drainage. In contrast, clayey soils may exhibit higher nutrient-holding capacity and microbial colonization but may hinder oxygen diffusion and leachate flow. The success of biostimulant application in these soils depends on tailoring amendment forms (*e.g*., slow-release *vs* soluble), dosage, and application method to soil porosity and water-holding characteristics. Although a geographic map of region-specific biostimulants was considered, it was omitted due to format limitations. Instead, region-specific examples have been highlighted in-text to illustrate the influence of local materials on remediation success.

Contaminant type and depth vary significantly between sites. For example, light petroleum hydrocarbons such as diesel or gasoline are more mobile and bioavailable than heavier fractions like crude oil or bitumen, which tend to adsorb tightly to soil particles and resist microbial attack. Sites near refineries or industrial plants may present mixed contaminants and demand multifaceted biostimulant formulations. In contrast, agricultural zones affected by fuel leaks may require simpler, organic-matter-based amendments with native microbial reinforcement.

Effectively addressing this spatial variability requires tailoring biostimulant formulations to the site’s environmental and soil-specific characteristics. Several case studies have demonstrated that adapting biostimulant formulations to local soils and climates—such as using sugarcane bagasse in Brazil or rice husk compost in Southeast Asia—can significantly enhance microbial activation and TPH removal efficiency. Additionally, integrating local microbial inocula or indigenous plant-assisted rhizoremediation with biostimulation can align treatment with endemic ecological conditions. Future work should prioritize regionally distributed field trials under varying climatic and soil conditions. These efforts can inform the creation of a globally adaptable, climate-responsive biostimulation protocol library for consistent and scalable implementation.

Recognizing and addressing these spatial and regional factors will improve the reproducibility, scalability, and sustainability of biostimulation technologies. Future research should include more comparative field trials across diverse geographic zones, with the goal of building a climate- and soil-type–responsive biostimulation protocol library to guide practitioners globally.

## Summary and outlook of the biostimulation approach

**Biostimulation is a sustainable and low-cost strategy** for remediating petroleum hydrocarbon-contaminated soils by activating indigenous microbial communities.

**Comparison of bioremediation methods:**

• *Biostimulation* uses native microbes and requires no external inoculation, focusing instead on nutrient amendment.
• *Bioaugmentation* introduces exogenous bacteria, requiring compatibility with local conditions.

**Key advantages of biostimulation:**

• Utilizes easily available materials.
• Reduces the chances of causing secondary pollution.
• Particularly effective in nutrient-deficient soils.

**Types of biostimulants:**

• **Organic fertilizers** (*e.g*., compost, agricultural/domestic waste):
  - Cost-effective and readily available.
  - Serve dual roles: waste management and soil amendment.
• **Mineral fertilizers:**
  - Provide precise nutrient ratios.
  - Often combined with other methods.
  - Higher cost than organics.
• **Biochar:**
  - Multifunctional, improves sorption and microbial support.
  - Typically used in combined remediation.
• **Plants (Phytoremediation):**
  - Long-term solution.
  - Effective when integrated with microbial or nutrient-based biostimulation.

**Challenges in field application:**

• Selection of appropriate biostimulants depends on:
  - *TPH degradation efficiency*
  - *Local availability and cost*
  - *Soil properties and contamination type*
  - *Required treatment duration*

**Outlook for future research:**

• Emphasis should shift to **combined biostimulant strategies** that leverage synergistic effects.
• Flexible combinations of materials may offer enhanced performance over single-agent treatments.

**Importance of microbial synergy:**

• Indigenous microbes act as functional consortia.
• Community-level interactions drive effective degradation.

**Recommendations for future work:**

• Standardize biostimulant selection based on regional and soil-specific parameters.
• Incorporate **modern microbial monitoring tools** (*e.g*., metagenomics) to:
  - Track community dynamics.
  - Optimize interventions in real time.
• Develop **adaptive and site-responsive biostimulation protocols** for field-scale implementation.

### Future research directions

Biostimulation continues to emerge as a promising and eco-friendly strategy for the remediation of petroleum hydrocarbon-contaminated soils. However, several knowledge gaps and implementation challenges remain. To enhance scalability, reproducibility, and practical impact, future research should prioritize the following four directions:

**1. Standardized nutrient regimes (C:N:P Ratios):**

A critical need exists for the standardization of biostimulation formulations and nutrient regimes, particularly with respect to carbon:nitrogen:phosphorus (C:N:P) ratios that optimize microbial hydrocarbon degradation. Inconsistent application rates and formulations across studies hinder reproducibility and field translation. Developing soil-type–specific or contaminant-specific nutrient guidelines will improve treatment predictability and consistency.

**2. *In-situ* monitoring systems:**

The development of real-time, field-deployable monitoring tools is essential for tracking remediation progress and enabling dynamic adjustment. Although molecular tools such as qPCR and metagenomics provide valuable insights into microbial dynamics, they are often restricted to laboratory settings. Emerging biosensor technologies, including microbial reporter systems and enzyme-based detectors, can facilitate on-site detection of hydrocarbons and microbial activity. When integrated with mobile data collection platforms or telemetry, these systems can support adaptive biostimulation management in the field.

**3. Cross-disciplinary collaboration:**

Achieving effective biostimulation requires collaborative input from soil scientists, microbiologists, geotechnical engineers, and environmental policymakers. Interdisciplinary frameworks are necessary to align microbiological insights with geotechnical and environmental objectives. For example, microbial data can inform models predicting soil strength recovery, while geochemical assessments can be used to tailor biostimulant selection and application strategies.

**4. Predictive modeling and artificial intelligence (AI):**

Computational modeling and machine learning offer promising approaches to simulate and optimize biostimulation outcomes under diverse environmental conditions. Predictive algorithms that integrate climatic data, soil properties, microbial community profiles, and contaminant characteristics can support site-specific remediation planning. These tools are especially valuable in data-scarce or resource-limited regions where trial-and-error strategies are impractical.

In summary, by advancing standardization of nutrient regimes, developing *in-situ* monitoring systems, fostering interdisciplinary collaboration, and implementing predictive modeling techniques, biostimulation can transition from experimental application to a robust, field-proven, and globally scalable approach for sustainable soil remediation.

## Conclusion

Biostimulation is increasingly recognized as a practical and environmentally sustainable strategy for remediating petroleum hydrocarbon-contaminated soils. By enhancing indigenous microbial activity through the addition of targeted nutrients or organic amendments, it offers advantages over traditional physical and chemical remediation methods. These advantages include cost-effectiveness, minimal secondary pollution, and the use of locally available materials, making it suitable for large-scale and *in situ* applications.

This review has examined the key mechanisms by which biostimulation enhances biodegradation. These include improving nutrient balance, modifying soil structure, and increasing microbial diversity. A wide range of biostimulants such as agricultural residues, domestic waste, mineral fertilizers, biochar, and plants has demonstrated considerable potential. The use of synergistic combinations of biostimulants has often produced more effective outcomes compared to single treatments, emphasizing the importance of integrated and context-specific approaches.

Despite these strengths, challenges remain in optimizing the application of biostimulation. Key issues include selecting the appropriate type and dosage of biostimulants, managing variability in soil and microbial characteristics across different sites, and maintaining stable remediation conditions over time. Treatment duration may also vary depending on contaminant properties, environmental conditions, and soil composition.

To advance the field, future studies should focus on creating standardized protocols for biostimulant application. Additional priorities include the development of real-time monitoring systems, long-term ecological evaluations, and predictive modeling techniques. Collaborative research involving soil scientists, microbiologists, and environmental engineers will be essential to ensure broader adoption and improved outcomes.

With continued research and technological development, biostimulation has strong potential to serve as a key strategy in global efforts to remediate petroleum-contaminated soils and restore environmental health.

## Supplemental Information

10.7717/peerj.19991/supp-1 Supplemental Information 1 Biochar, through adsorption and its porous structure, improves soil properties and provides nutrients. The graphical abstract shows that It creates a favorable habitat for microorganisms, enhancing their colonization and metabolic activity. These effects promote the breakdown of petroleum hydrocarbons in contaminated soil. Ultimately, this leads to a significant reduction in petroleum hydrocarbon content.

## Additional Information and Declarations

### Data Availability

The following information was supplied regarding data availability:

This is a literature review.
