# Supplementary figures and images for: Progress in biostimulation-based remediation of TPH-contaminated soils: a comprehensive review
Source: PeerJ. 2025 Sep 8;13:e19991. doi: 10.7717/peerj.19991 (PMC12424616; doi:10.7717/peerj.19991)

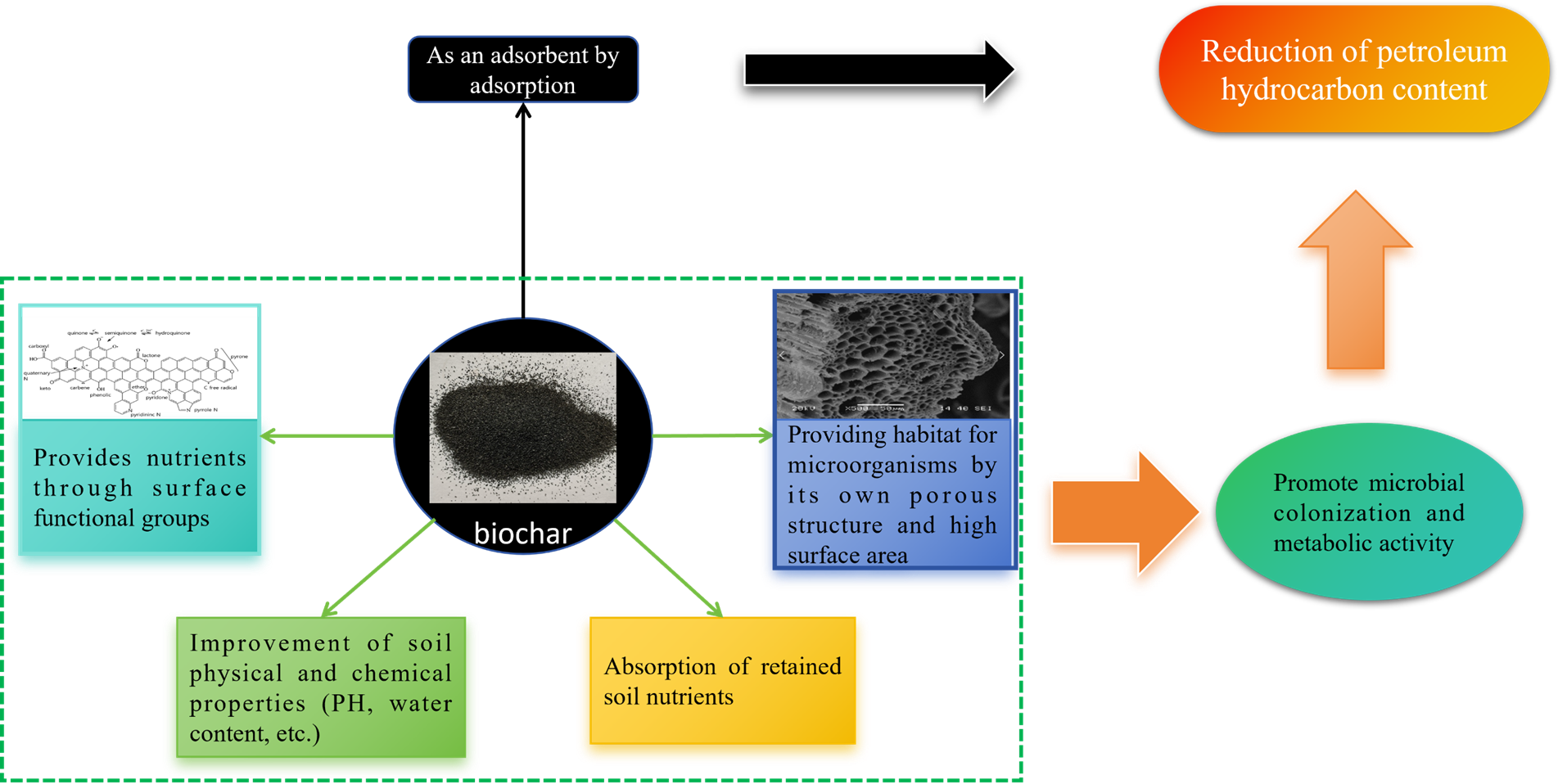

Supplement: Supplemental Information 1 — The graphical abstract shows that It creates a favorable habitat for microorganisms, enhancing their colonization and metabolic activity. These effects promote the breakdown of petroleum hydrocarbons in contaminated soil. Ultimately, this leads to a significant reduction in petroleum hydrocarbon content. [file peerj-13-19991-s001.png]
